# Supplementary material for: Genetic Inactivation of Peroxiredoxin-I Impairs the Growth of Human Pancreatic Cancer Cells
Source: Antioxidants (Basel). 2021 Apr 8;10(4):570. doi: 10.3390/antiox10040570 (PMC8068151; doi:10.3390/antiox10040570)
Supplement: Supplementary file 1 [file antioxidants-10-00570-s001.pdf]

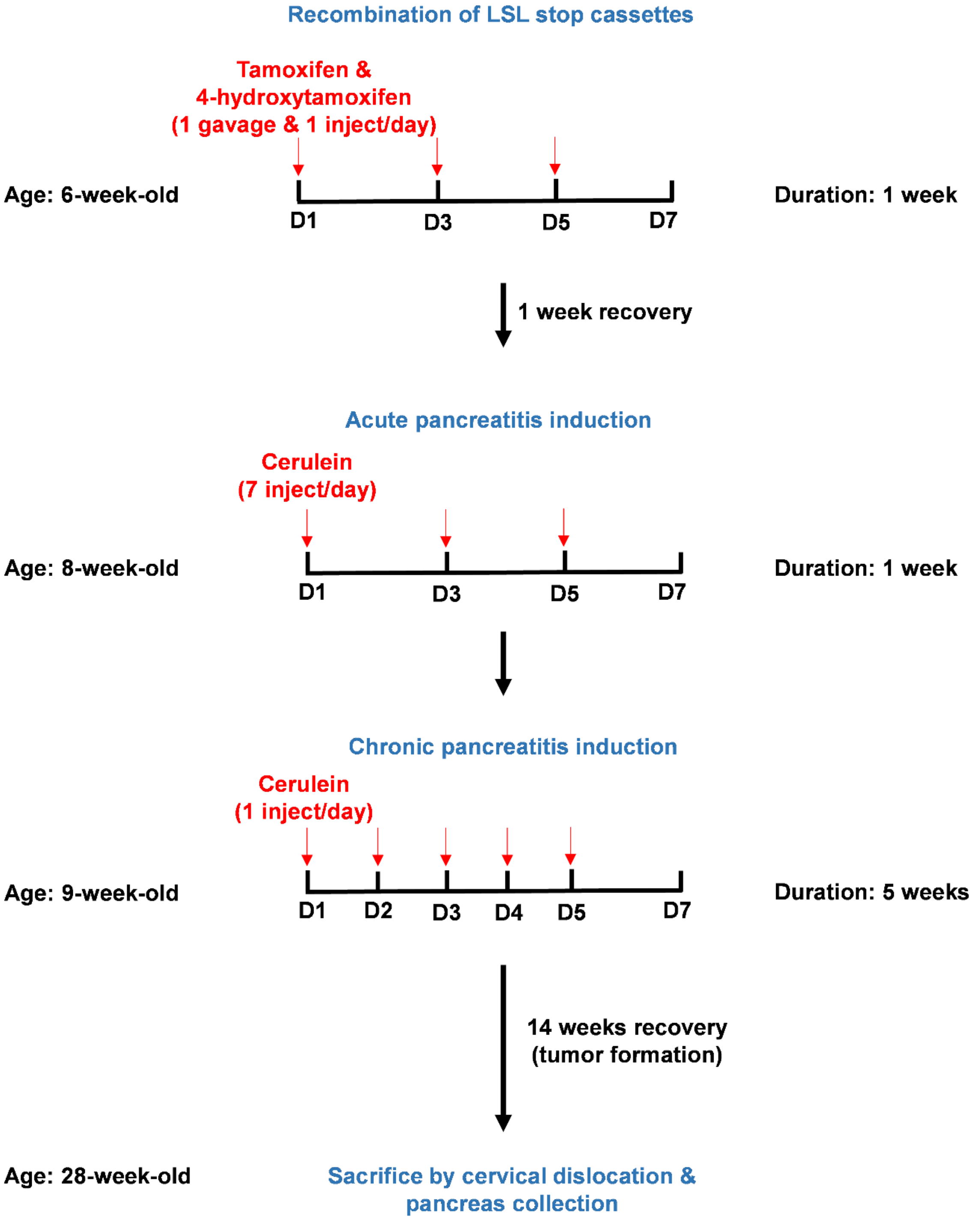

Figure S1. Illustration of the used tamoxifen and cerulein regimens in *ElastaseCreER-LSLKras^{G12D}-p53^{R172H}* (*KPE*) mice. Adult 6-week-old *KPE* mice were first treated once a day with tamoxifen (gavage) and 4-hydroxytamoxifen (subcutaneous injection), at days (D) 1, 3 and 5 (red arrows), to recombine the LSL stop cassettes and allow the expression of *Kras^{G12D}* and *p53^{R172H}* from their respective endogenous locus. After tamoxifen injections, mice were let recover for 1 week. Next, mice started an acute pancreatitis regimen of 1 week consisting of 7 dailies intraperitoneal cerulein injections (1 injection/hour), at days (D) 1, 3 and 5 (red arrows); the dose of each cerulein injection was 125 µg/Kg in a volume of 100-150 µl (diluted in PBS). At the end of acute treatment, mice started a chronic pancreatitis regimen of 5 weeks. Each week, mice received one daily intraperitoneal cerulein injection at days (D) 1, 2, 3, 4 and 5; the dose of each cerulein injection was 125 µg/Kg in a volume of 100-150 µl (diluted in PBS). At the end of chronic treatment, mice were kept for 14 additional weeks to allow tumor formation and were then sacrificed by cervical dislocation.

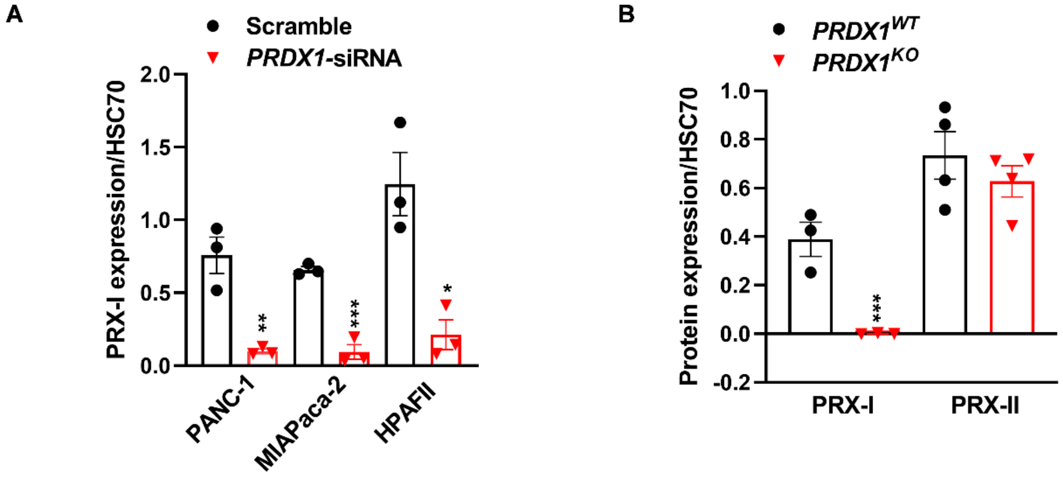

Figure S2. Peroxiredoxin-I (PRX-I) expression in human pancreatic ductal adenocarcinoma cells following genetic inactivation of *Peroxiredoxin-1* (*PRDX1*) gene by small interfering RNA (siRNA) or clustered regularly interspaced short palindromic repeats/CRISPR-associated protein 9 (CRISPR/Cas9). (**A**) Densitometry quantification for western blots shown in **Figure 2A**. Heat-shock cognate 70 (HSC70) was used as a loading control. Experiments were performed on three independent passages (n=3). (**B**) Densitometry quantification for western blots shown in **Figure 2F**. HSC70 was used as a loading control. Experiments were performed on at least three independent passages (n=3). Data are mean ± SEM. Statistical significance was tested by Student t-test (* *P*<0.05, ** *P*<0.01, *** *P*<0.001).

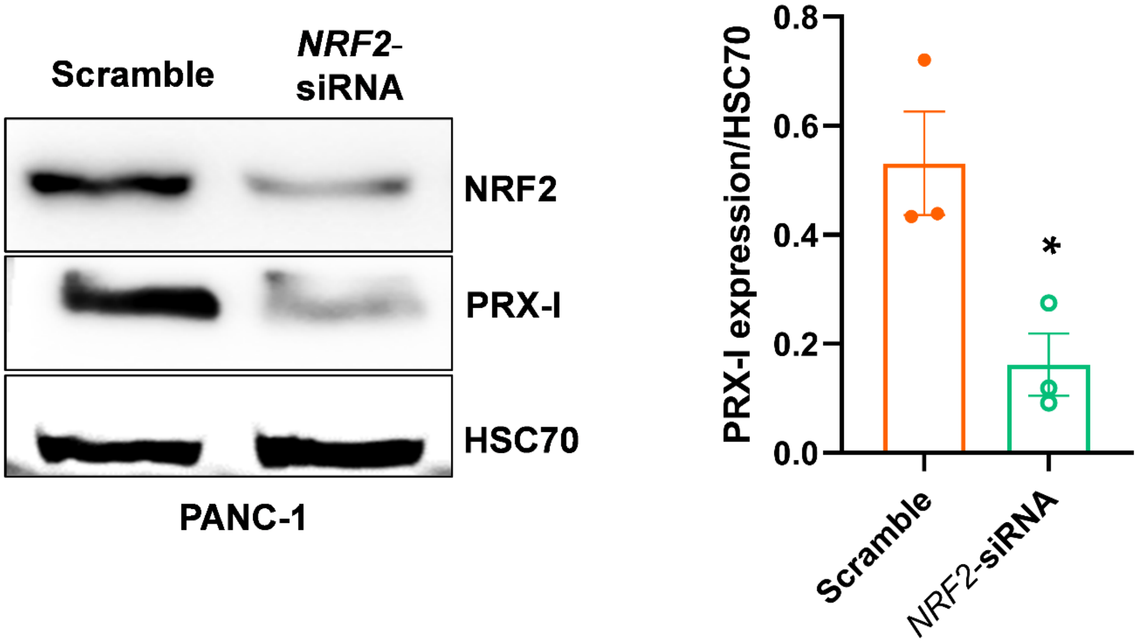

Figure S3. Nuclear factor erythroid 2-related factor 2 (NRF2) regulates the expression of Peroxiredoxin-I (PRX-I) in human pancreatic ductal adenocarcinoma cells. Western blot analysis on PANC-1 cells transfected with Scramble or specific *NRF2*-siRNA. The corresponding densitometry analysis is also available; experiments were performed on three independent cultures (n=3). Data are mean ± SEM. Statistical significance was tested by Student t-test (**P*<0.05).

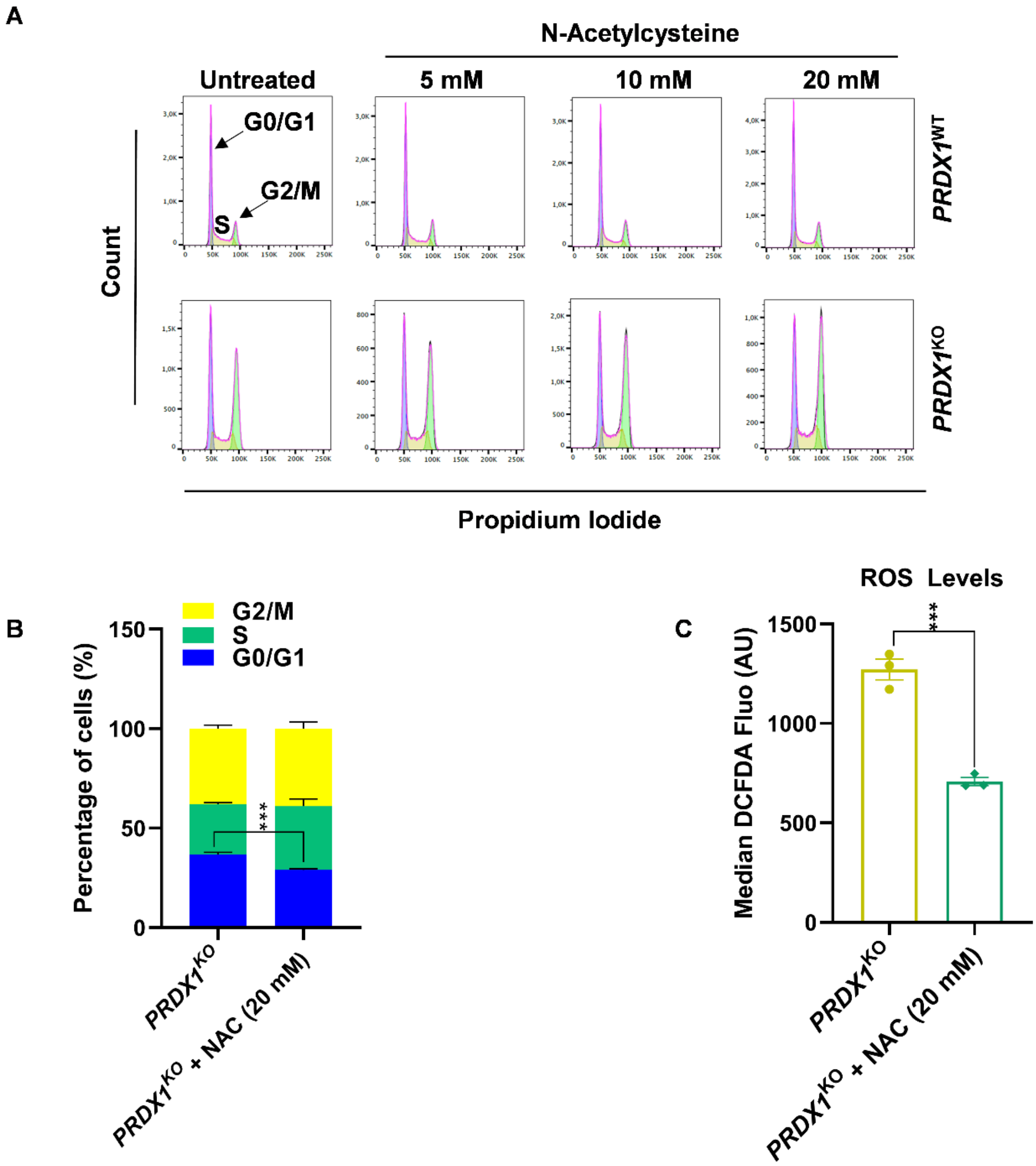

Figure S4. N-Acetylcysteine (NAC) treatment does not reverse cell cycle blockade at G2/M in human pancreatic ductal adenocarcinoma cells. (**A**) Typical Plots from FACS cell cycle analysis on *PRDX1^{WT}* and *PRDX1^{KO}* lines untreated or treated with different NAC doses for 16 hours. (**B**) Percentage of *PRDX1^{KO}* cells in the different cell cycle phases following NAC treatment (20 mM) for 16 hours; experiments were performed on three independent cultures (n=3). (**C**) Reactive oxygen species (ROS) levels, determined by FACS, in *PRDX1^{KO}* cells treated or not with NAC (20 mM) for 16 hours; measurements were performed on three independent cultures (n=3). Data are mean ± SEM. Statistical significance was tested by Student t-test (****P*<0.001).

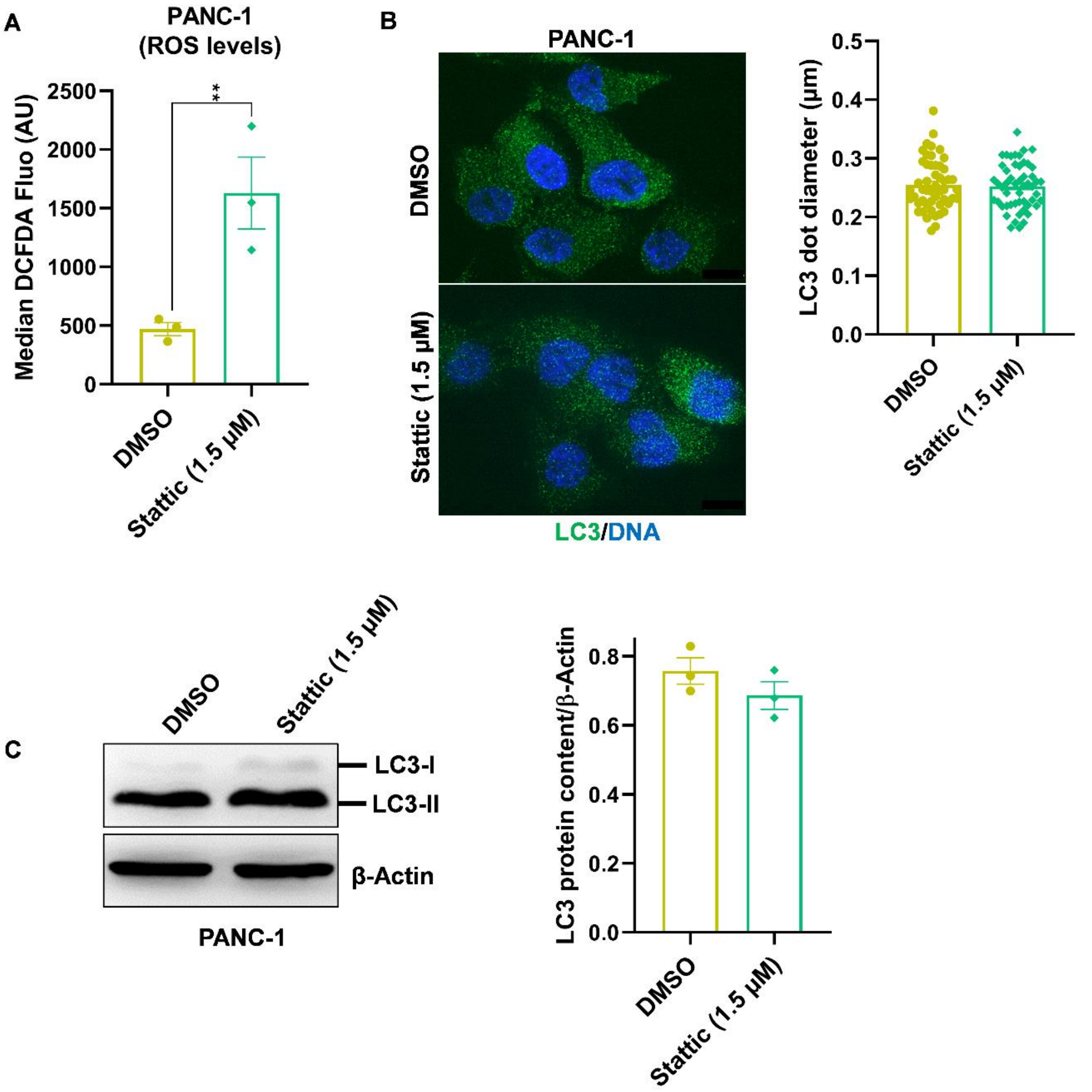

Figure S5. Inhibition of Signal transducer and activator of transcription 3 (STAT3) induces reactive oxygen species (ROS) production without impacting the autophagic flux in human pancreatic ductal adenocarcinoma cells. (**A**) Reactive oxygen species (ROS) levels, determined by FACS, in PANC-1 cells treated with DMSO (0.015%) or Stattic (1.5 μM) for 16 hours; measurements were performed on three independent cultures (n=3). AU: Arbitrary Units. (**B**) Representative confocal images of Microtubule-associated protein 1A/1B-light chain (LC3) immunolabeling on PANC-1 cells treated with DMSO (0.015%) or Stattic (1.5 μM) for 16 hours; immunolabeling was performed on two independent cultures (n=2) and 51-to-52 cells were selected randomly from confocal images to measure LC3 dots diameter. (**C**) Western blots for LC3 and the corresponding densitometry quantification performed on PANC-1 cells treated with DMSO (0.015%) or Stattic (1.5 μM) for 16 hours. β-Actin was used a loading control. Western blots were performed on three independent cultures (n=3). Data are mean ± SEM. Statistical significance was tested by Student t-test (***P*<0.01).
